# Supplementary material for: Intertwined Functions of Separase and Caspase in Cell Division and Programmed Cell Death
Source: Sci Rep. 2020 Apr 9;10:6159. doi: 10.1038/s41598-020-63081-w (PMC7145830; doi:10.1038/s41598-020-63081-w)

Intertwined Functions of Separase and Caspase in Cell Division and Programmed Cell Death

Pan-Young Jeong[1], Ashish Kumar[1], Pradeep Joshi[1], and Joel H. Rothman[1]*

Supplemental material

Supplemental Figure S1. Compromised SEP-1 separase function results in extra surviving nuclei

L1 larvae stained with DAPI show presence of extra nuclei (arrows) in the anterior pharynx of either *sep-1(e2406)* alone (middle panel) or in conjunction with the weak reduction of function *ced-3(n2443)* allele (bottom panel), as compared to the laboratory reference strain N2 (top panel)

Supplemental Figure S2. ***ced-3* enhances reduction of function defects of *sep-1* RNAi treated animals**

(A) Embryonic lethality of embryos from parents fed with HT115 *E. coli* strain expressing dsRNA against *sep-1* (from the Ahringer RNAi library collection) diluted with bacteria carrying an empty vector. (B) % of males among the surviving progeny of L4 parents fed diluted *sep-1* RNAi bacteria.

Supplemental movies

Movie S1. *ced-3(n717); ruIs32*. Normal chromosome segregation in *ced-3* mutant strain expressing histone 2B-GFP fusion protein

Movie S2. *sep-1(e2406); ruIs32*. Normal chromosome segregation in *sep-1* mutant strain expressing histone 2B-GFP fusion protein at the permissive temperature of 19.5°C.

Movie S3. *sep-1(e2406); ced-3(n717); ruIs32.* Failure of egg shell formation and chromosome segregation at the permissive temperature of 19.5 ±0.2°C

Movie S4 *sep-1(e2406); ced-3(n717); ruIs32.* No apparent defects in either egg shell formation or chromosome segregation at 18.2 ±0.2°C

Supplementary Figures

Figure S1 Extra surviving pharyngeal cells in *sep-1(e2406)*

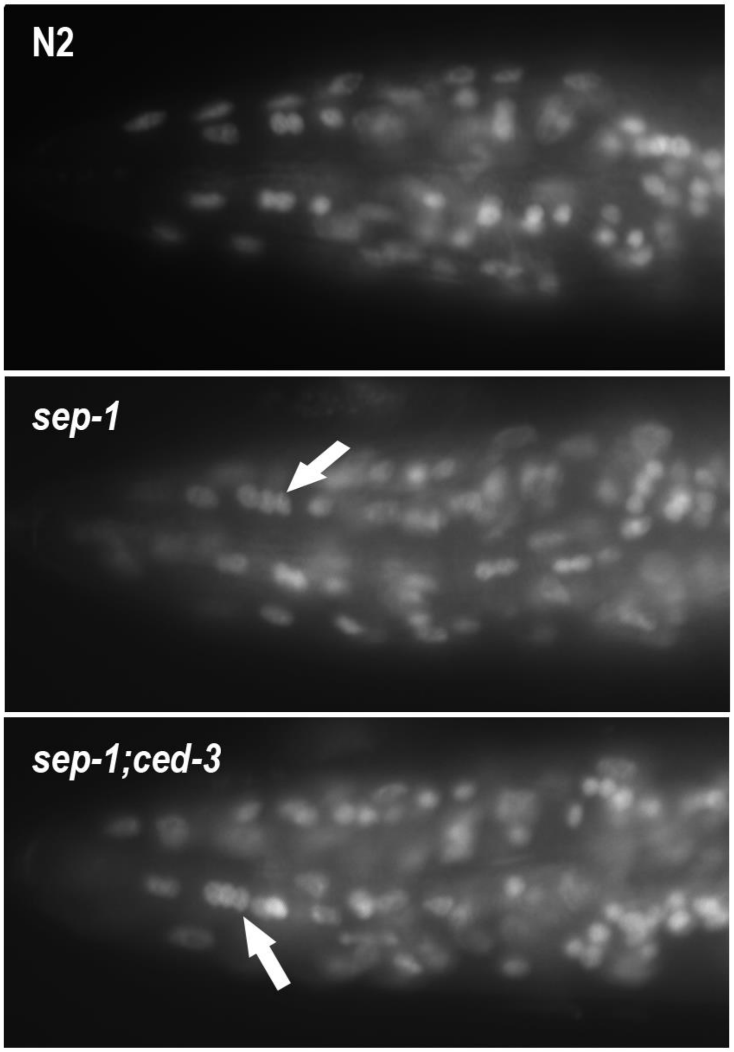

Figure S2. ***ced-3*** **enhances reduction of function defects of** ***sep-1*** **RNAi treated animals**

A

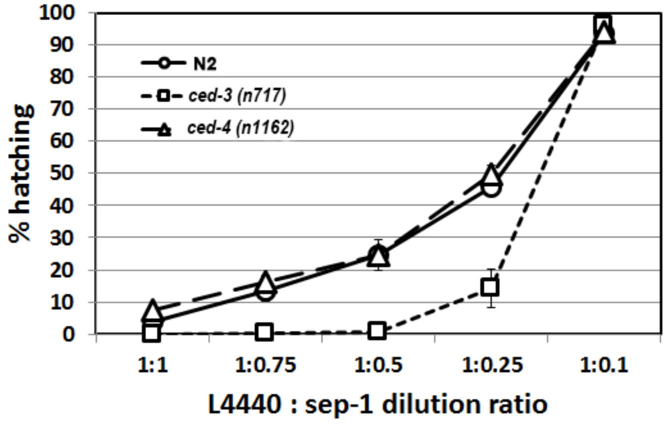

B

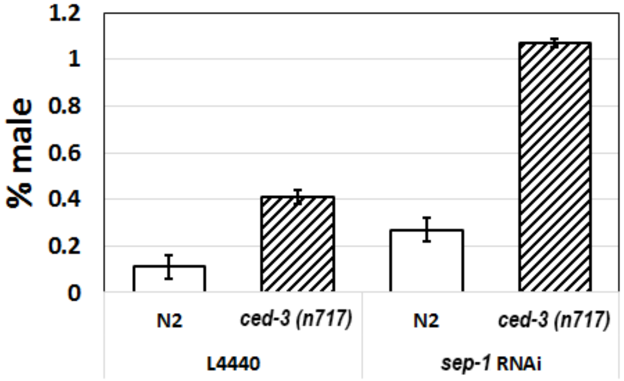

Supplement: Supplementary file 1 — Supplementary Information. [file 41598_2020_63081_MOESM1_ESM.pdf]
